# Intermediate-dose TBI/fludarabine conditioning for allogeneic hematopoietic cell transplantation in patients with cutaneous T-cell lymphoma

**DOI:** 10.1038/s41409-025-02796-8

**Published:** 2026-01-20

**Authors:** Isabelle Krämer, Laila König, Arne Brecht, Jessica C. Hassel, Thomas Luft, Ute Hegenbart, Tanja Eichkorn, Peter Stadtherr, Carsten Müller-Tidow, Peter Dreger

**Affiliations:** 1 Internal Medicine V, Hematology, Oncology and Rheumatology, Heidelberg University Hospital, Heidelberg, Germany; 2 Department of Radiation Oncology, Heidelberg University Hospital, Heidelberg, Germany; 3 Internal Medicine V, Hematology, Oncology and Palliative Medicine, Klinikum Darmstadt, Darmstadt, Germany; 4 Heidelberg University, Medical Faculty Heidelberg, Department of Dermatology and National Center for Tumor Diseases (NCT), NCT Heidelberg, a partnership between DKFZ and Heidelberg University Hospital, Heidelberg, Germany

**Keywords:** T-cell lymphoma, Stem-cell therapies

## To the Editor:

Allogeneic hematopoietic cell transplantation (alloHCT) is an effective treatment for patients with relapsed/refractory advanced mycosis fungoides and Sezary syndrome (MF/SS) [1, 2]. However, the optimum strategy of alloHCT for MF/SS, in particular choice of conditioning regimen and timing of transplantation, remains unclear [3]. Recently, we have described favorable outcomes of alloHCT using a conditioning regimen based on intermediate-dose total body irradiation (TBI) and fludarabine in patients with peripheral T-cell lymphoma (PTCL) [4]. Here, we report our experience with this approach in MF/SS.

## Patients and methods

Eligible for this retrospective single-center analysis were all adult patients who had undergone a first alloHCT for MF/SS from a matched related or unrelated donor in our institution and received conditioning with intermediate-dose TBI. According to international guidelines, patients had to have advanced disease (EORTC stage IIB or higher) and failed at least one line of systemic therapy. Patients with stage IV disease, large cell transformation, and necrotizing tumors were allowed to undergo alloHCT as part of the first systemic treatment line [3, 5]. Total body irradiation (TBI) was given in 3–4 fractions at 2 Gy on two consecutive days. Further details on conditioning and graft-versus-host disease (GVHD) prophylaxis have been described previously [4].

Baseline characteristics, treatment details, and outcome data were extracted from electronic chart review. The study was performed in accordance with the Declaration of Helsinki. All patients provided written informed consent to data collection and scientific evaluation before admission to alloHCT. Data analysis was approved by the institutional review board.

Kaplan-Meier product-limit estimates were used for assessing survival probabilities. Graft-versus-host disease and relapse/progression-free survival (GRFS) was calculated according to the EBMT definition of “refined GRFS”, counting acute GVHD III-IV, severe chronic GVHD, relapse/progression, and death as events [4]. Survival curves were compared using log-rank test. Estimates of chronic GVHD, non-relapse mortality (NRM), and relapse incidence (REL) were analyzed using cumulative incidence rates in a competing risk framework. GraphPad Prism software (release 9.0; San Diego, CA) was used for calculations and image. Data were analyzed as of August 5, 2025.

## Results

Altogether, 16 consecutive patients with MF/SS (MF 10, SS 6) received an alloHCT after TBI/fludarabine-based conditioning as institutional treatment standard between April 2014 and February 2025. Median age at transplantation was 57 (22–72) years, and the median time between diagnosis and alloHCT 14 (6–107) months. Disease stage was IIB in 5 patients (31%), IVA2 in 9 patients (56%), and IVA1 and IVB in one patient each. Six patients (38%) had transformed disease, and one patient necrotizing MF. The median number of systemic pretreatment lines was 2 (1–5), with 7 patients (44%) having received only one line. 10 patients (63%) had been exposed to one or multiple monoclonal antibodies (brentuximab vedotin, 9; mogamulizumab, 3; alemtuzumab, 2; checkpoint inhibitor, 1). With a minimum wash-out time of 6 months, none of the four patients receiving mogamulizumab and checkpoint inhibitor, respectively, developed acute or chronic GVHD. Disease status at alloHCT was CR/PR in 7 patients (44%), stable disease in 5 patients (31%), and progressive disease in 4 patients (25%). All patients underwent alloHCT in a good performance status (ECOG 0, 13; ECOG 1, 3) with low comorbidity load (HCT-CI 0, 13; HCT-CI 1–2, 3). The TBI target dose of 8 Gy was met in 15 patients (94%) whereas a single patient received 6 Gy only (Table S1).

**Fig. 1:**
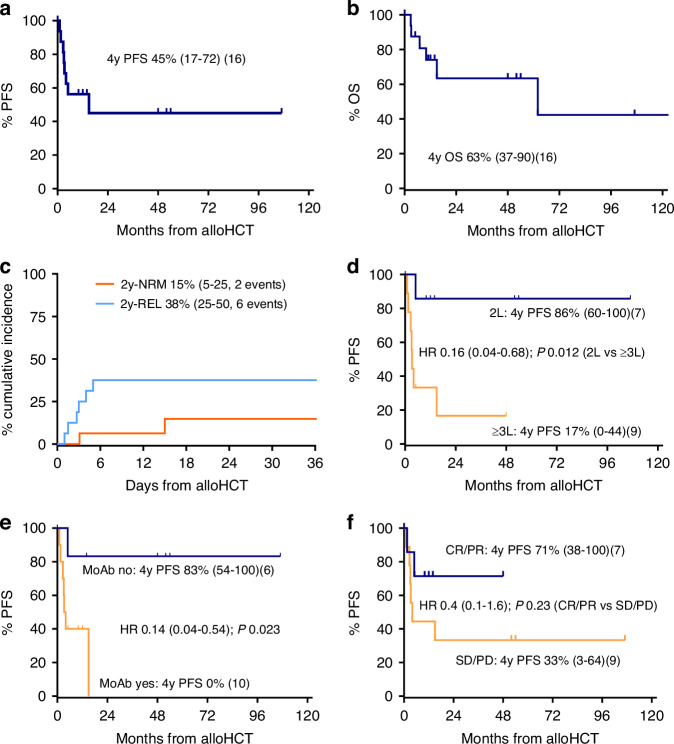
Survival outcomes. Progression-free survival (PFS) (**a**), overall survival (OS) (**b**), relapse incidence (REL) and non-relapse mortality (NRM) (**c**), PFS by treatment line (**d**), PFS by monoclonal antibody exposure (**e**), and PFS by disease status at transplantation (**f**). alloHCT allogeneic hematopoietic cell transplantation, CR complete response, L line, MoAb monoclonal antibody, PD progressive disease, PR partial response, SD stable disease.

With a median follow-up of survivors of 31 (5–133) months, 6 patients have died, 4 of MF/SS, and 2 for non-relapse causes (macrophage activation syndrome and atypical pneumonia, respectively). The cumulative incidence of NRM at 2 years was 15% (95% confidence interval (95%CI) 5–25%) (Fig. 1c). Altogether, 6 (38%) relapse/progression events occurred, all within the first 6 months post-transplant. One could be durably salvaged by radiotherapy, and one is currently treated with donor lymphocyte infusions. REL, progression-free survival (PFS), and overall survival (OS) at 3 years were 38% (95%CI 25–50%), 45% (95%CI 17–72%), and 63% (95%CI 37–90%), respectively (Fig. 1a–c).

Notably, receiving alloHCT as part of the first systemic treatment line was associated with significantly favorable PFS (86% at 3 years), as was not being exposed to monoclonal antibodies (83% at 3 years). In addition, patients undergoing transplantation with responsive disease showed a numerically substantially better PFS (71% at 3 years vs 33% in non-responders) (Fig. 1d–f). In contrast, age, stage, SS, disease transformation, and time from diagnosis were not significantly associated with PFS in this small sample.

Acute toxicity was modest with grade 3 mucositis recorded in 6 patients (38%) and a median duration of hospitalization after alloHCT of 20 (13–27) days. All patients had stable neutrophil engraftment, and durable full hematopoietic donor chimerism was achieved in 14 patients (88%) after 38 (20–80) days. One of the remaining two patients died early because of NRM, and the other is currently receiving donor lymphocytes for chimerism completion while being in ongoing CR. Acute GVHD occurred in only 2 patients (grade II and grade III, respectively). Chronic GVHD occurred in 4 of 13 patients at risk (31%), but was severe in only 1 of them. The cumulative incidence of chronic GVHD (using death and relapse as competing risks) at 24 months was 22%; and 4-year GRFS was 38% (95%CI 9–66%). No relapse/progression event occurred after the onset of chronic GVHD.

## Discussion

Although small, this series documents that intermediate-dose TBI is a safe and effective conditioning regimen also for MF/SS allotransplants. Similar to our experience in PTCL, acute toxicity and risks of acute and chronic GVHD were modest, translating into low NRM. With 38% at 3 years, relapse risk appeared to be higher than observed in PTCL (24% at 3 years), largely caused by poorer disease control in patients with stable or progressive MF/SS at alloHCT [4]. Nevertheless, our PFS data compare favorably to those of larger prospective and retrospective multicenter studies reporting 3-year PFS rates of 19–37% [1, 6, 7]. Two small retrospective single-center series reported similarly good disease control as in our study with conditioning based on even lower TBI doses (2–6 Gy), and a Japanese registry study did not find survival differences between patients receiving low- and high-dose TBI, respectively [6, 8, 9]. These observations may suggest that there is no TBI dose effect in MF/SS, however, since early progression is the main driver of alloHCT failure in MF/SS as documented also in the present series, there is some rationale to increase conditioning intensity as long as good tolerability is maintained.

Although our sample size is too small for meaningful risk factor analyses, the favorable outcome of patients receiving alloHCT as part of the first systemic treatment line and those without prior exposure to monoclonal antibodies was intriguing. Since these two parameters were highly correlated, however, the significance of each of them remains speculative, albeit there is some plausibility for considering treatment line as the more important driver here [7, 10, 11].

In conclusion, this study demonstrates that intermediate-dose TBI/fludarabine-based conditioning provides promising outcomes of alloHCT also in MF/SS with a favorable toxicity/efficacy ratio although anti-lymphoma activity appears to be limited in patients with refractory disease. An auxiliary finding was the favorable prognosis of patients transplanted after only one line of systemic pretreatment and those who were monoclonal antibody-naïve, warranting substantiation in larger studies.

## Supplementary information

Table S1

## Data Availability

The datasets generated during and/or analysed during the current study are available from the corresponding author on reasonable request.
