# Supplementary material for: Intermediate-dose TBI/fludarabine conditioning for allogeneic hematopoietic cell transplantation in patients with cutaneous T-cell lymphoma
Source: Bone Marrow Transplant. 2026 Jan 20;61(4):484–6. doi: 10.1038/s41409-025-02796-8 (PMC13056524; doi:10.1038/s41409-025-02796-8)
Supplement: Supplementary file 1 — Table S1 [file 41409_2025_2796_MOESM1_ESM.docx]

**Supplemental Appendix**

**Content**

**Table S1:** Patient characteristics at baseline ……………………………………………………………….……..… 2

**Table S1:**

Patient characteristics at baseline.

|  | **n=16** |
| --- | --- |
| MF  SS | 10 (62.5%)  6 (37.5%) |
| Median age (years) | 57 (22-72) |
| Median time between diagnosis an alloHCT (months) | 14 (6-107) |
| Disease stage  IIB  IVA1  IVA2  IVB | 5 (31%)  1 (6%)  9 (56%)  1 (6%) |
| Transformed disease | 6 (38%) |
| Median number of systemic pretreatment lines  1  2  ≥ 3 | 2 (1-5)  7 (44%)  5 (31%)  4 (25%) |
| Disease status  CR  PR  SD  PD | 2 (13%)  5 (31%)  5 (31%)  4 (25%) |
| Performance status  0  1  >1 | 13 (81%)  3 (19%)  0 (0%) |
| HCT-CI  0  1-2 | 13 (81%)  3 (19%) |
| TBI dose (Gy)  6  8 | 1 (6%)  15 (94%) |
| Donor  MRD  MUD | 3 (19%)  13 (81%) |

allogeneic hematopoietic cell transplantation (alloHCT), complete response (CR), hematopoietic cell transplantation comorbidity index (HCT-CI), mycosis fungoides (MF), fully matched related donor (MRD), fully matched unrelated donor (MUD), partial response (PR), progressive disease (PD), Sezary syndrome (SS), stable disease (SD), total body irradiation (TBI)
